# Teachers’ Perceptions and Position Regarding the Problem of Bullying and Its Socio-Educational Prevention

**DOI:** 10.3390/bs14030229

**Published:** 2024-03-12

**Authors:** Sara Martínez-Carrera, Cristina Sánchez-Martínez, Isabel Martínez-Carrera, Miguel Ángel Díaz Dieguez

**Affiliations:** 1 Didactic and School Organization Department, CEU Escuela Universitaria de Magisterio, 36214 Vigo, Spain; 2 Didactic and School Organization Department, Universidade de Vigo, 32004 Ourense, Spain; c.sanchez@uvigo.es (C.S.-M.); isabel.martinez.carrera@uvigo.es (I.M.-C.); 3 Independent Researcher, 11130 Cádiz, Spain; miguel.diaz.hugo@gmail.com

**Keywords:** social behaviours at school, learning behaviours, teacher behaviours, bullying, prevention, education, teachers, students

## Abstract

The problems that arise with coexistence between schoolchildren are a challenge when it comes to carrying out the teaching–learning process. In recent years, the presence of bullying has increased in schools. The aim of this study is to identify the perceptions of teachers regarding the problems of coexistence among schoolchildren, more specifically concerning bullying, as well as to identify their position regarding the prevention of bullying. Research was carried out using a mixed approach. A total of 225 education professionals from different schools in Spain took part. From the results and our conclusions, it should be noted that teachers consider themselves to have a high level of knowledge of the essential characteristics of the dynamics of bullying, of the actors involved, and of some preventive measures that can be carried out to avoid this type of behaviour as much as possible. On the other hand, a large number of teachers downplay the importance of the problem of coexistence in their fields, and/or are not able to identify it, which makes it impossible to act. Some schools hardly carry out any prevention work, which makes it difficult to eradicate bullying.

## 1. Introduction

The study of human behaviour in educational settings has great applications in different aspects of learning and teaching, such as how a teacher remains interested in teaching and how a learner engages in challenging behaviours during learning.

In this sense, the problems of coexistence among schoolchildren present themselves as a challenge when the teaching–learning process is carried out, and among them, it is worth mentioning the problems related to bullying.

Bullying is a form of aggression that occurs in the school environment. Specifically, bullying is defined as repeated aggressive behaviour by a perpetrator toward one or more of their classmates [1,2].

Bullying is a serious problem in schools around the world [3,4]. In recent decades, the prevalence and impact of bullying have been the subject of social concern. Therefore, the aim of this study is to identify the perceptions of teachers regarding the problems of coexistence among schoolchildren, more specifically bullying, as well as to identify their position regarding the prevention of bullying. In terms of research questions, the following questions were posed:

- RQ1. What are teachers’ perceptions regarding the problems of school coexistence, specifically bullying?
- RQ2. What is the position of teachers on the prevention of bullying?

There is much room for improvement in the actions taken by society, especially by education professionals regarding the problem of bullying, and this is applicable to a greater or lesser extent in all countries. The findings of this work may help to clarify the current situation of teachers regarding bullying in order to raise awareness for preventive behaviours/programmes.

### 1.1. Problems of Coexistence at School: Bullying

School coexistence refers to the relationships or interactions between different educational agents: teachers, students, and families, but it also alludes to society in general, as it is also involved in education in a more or less direct way [5]. More specifically, the main issues that have an impact on coexistence concern attention to diversity, self-esteem, collaboration, stress, and bullying. This is why school coexistence is a cross-cutting issue that has significant influence on aspects such as the performance of educational institutions, the bullying rate, students’ academic results, or teachers’ stress. Consequently, all of this has an impact on the school climate regardless of the level of education, which makes it even more important.

For the correct development of children and adolescents, it is important that there be an appropriate school climate and a positive environment that favours wellbeing. In this circumstance, it is where better academic results can be obtained, as well as fewer mental health problems and benefits to mental health such as working in an empathetic and respectful environment where relationships are healthy and positive. A positive school environment, free from bullying and discrimination, is fundamental for the healthy development of young people [6].

Bullying is a type of violence currently widely discussed worldwide [7,8,9]. This research only deals with school bullying, i.e., bullying that occurs in schools between individuals usually aged 4–18 years old. Bullying is understood as repeated acts of violence between peers, where there is an unequal power relationship, not including people in a couple or siblings. These aggressions can be physical, verbal, or virtual. This behaviour mainly occurs in school settings and can have long-term effects on the mental health and wellbeing of those affected [6,10,11,12,13].

Bullying is one of those circumstances where coexistence in a school is damaged. Studies have shown that during the COVID-19 pandemic, the lockdown of children at home resulted in less contact with their peers, which led to a decrease in incidents of bullying [14,15,16,17,18,19]. Bullying, nevertheless, is a serious problem that affects a large number of students around the world.

### 1.2. Actors Involved

Several parties are involved in bullying situations. On the one hand, students can have several roles, being victims, aggressors, or bystanders. In the first case, it can lead to problems such as low self-esteem, anxiety, or decreased scholastic performance [20]. In addition, victims of bullying are often less powerful than bullies or groups of bullies and feel that they cannot easily defend themselves. This may be due to a physical or social power imbalance [21]. On the other hand, the situation of bullies can also lead to relationship problems or antisocial behaviour. Bullies are individuals who intend to cause harm to their victims through their actions over a long period of time [22,23]. Bullies show low levels of kindness and conscientiousness, showing high levels of extraversion and neuroticism and, unexpectedly, lower levels of openness as well [24]. In the school context, bullying is a complex social phenomenon that often does not occur between a bully and victim in isolation. For example, individuals may be involved in bullying not only as bullies or victims but also as bystanders, defenders, or reinforcers [2,25].

Moreover, education professionals play an essential role in bullying prevention and intervention. Teachers and school staff must be trained to recognise the signs of bullying and act effectively [26]. There must be an appropriate and positive school environment. This is elementary for a climate of respect and coexistence that would not favour situations of inequality. Teachers can lead, together with the school management team, different actions that provide constructive communication, an intervention of individualised attention to problems of indiscipline, and a more cooperative atmosphere in the classroom in cases of bullying. Along these lines, teachers can favour the creation of spaces for student participation and protagonism that would allow for collective reflection on the difficulties encountered in order to maintain the proper functioning of the school routine [27,28].

The active participation of families in the school is of great importance for sealing their commitment and interest in their children, as their role is very relevant in school coexistence. In the case of conflict, it is important that families know how to solve problems correctly. For this, affective communication is fundamental, where social skills are valued and a comprehensive and interdisciplinary vision of the problem that has arisen is taken into account. Sometimes, victims find it difficult to talk to their parents because they perceive that they do not listen to them. They may also have little confidence in talking to their teachers [29]. Maintaining good family–school contact and relationships is extremely important. Olweus [6] suggests that parents should be active participants in their children’s education and wellbeing, both at home and at school. Other studies have shown that perceived social support from family [30,31] and friends [30] is crucial. Several studies found significantly less bullying victimisation when children are listened to by their parents [32,33]. It was also shown that being listened to by parents is a strong protective factor, as it is important that children and adolescents feel adequately listened to in order to protect them from being victimised [34,35]. No less important is the weight of the school community or society in general, as preventing bullying situations, or becoming involved in bullying, is not only something that concerns the aforementioned groups.

### 1.3. Preventive Measures

Bullying is a social concern of great public health relevance because of the social and developmental impact on those involved [36,37,38]. It is undeniable that bullying can have negative or even harmful consequences for both victims and aggressors. It is therefore necessary to implement strategies to prevent the incidence of bullying. Effective strategies over time remain difficult, despite the consistent literature on the subject [39,40]. The existence of awareness and prevention programmes is important. Bullying prevention programmes have proven to be effective in reducing the incidence of bullying. Focusing on changes at school, at the classroom and individual level, should include awareness raising and the training of teachers and students [6,41]. These interventions should be targeted at various groups so that they have the tools to prevent, recognise and act [42,43]. Not only professionals but also families and the community should be involved in order to develop a more holistic approach to address the situation in an appropriate way. The importance of constant communication between the school and families to detect and manage cases of bullying is essential.

For their part, there must also be policies and protocols for action. Educational institutions should be clear about the procedures for prevention and action. Schools with well-defined policies that are known by the entire educational community have lower rates of bullying. In this regard, it is suggested that interventions that focus on emotional development and the rehabilitation of bullies are effective in preventing recidivism [44,45].

While “traditional” bullying in offline contexts remains a problematic phenomenon [46], the spread of online forms of bullying is now also considered a pressing issue, especially considering the fact that cyberbullying has been shown to be highly correlated with offline bullying but has unique characteristics and worrying prevalence rates [47]. When discussing the use of technology for social networking, it is important to highlight that online bullying, including cyberbullying, should be addressed in prevention programmes [48,49]. Research findings suggest that cyberbullying intervention programmes are effective in reducing both the perpetration and victimisation of cyberbullying. Several studies reveal the importance of anti-cyberbullying programmes, as they can reduce cyberbullying perpetration by approximately 10–15% and cyberbullying victimisation by approximately 14% [21,50,51].

Bullying is undoubtedly a major problem that needs a general and comprehensive perspective. For this reason, it is important to count on the collaboration and support of families, education professionals, and students. If students live in a welcoming, calm, and safe environment, it is more difficult for bullying to occur, and if it does occur, it is easier to count on the participation, sensitivity, and education of the community. Both prevention and intervention are key when it comes to bullying. We must all work together in the quest for a fairer, more tolerant, and respectful society.

## 2. Materials and Methods

The research presented utilised a mixed methodology, in particular a qualitative treatment based on a narrative approach developed under a multiple case study. Qualitative methodology allows for the description and understanding of the reality analysed, taking into account the peculiarities of the study context [52]. Qualitative researchers maintain the individuality of each participant instead of reaching large samples [53]. This form of research is used to collect data from the real world without pre-established responses and then interpret the meaning that individuals associate with the particular situation [54,55].

Through identification, description, analysis, interpretation, and explanation, it improves the understanding of reality [56]. In this study, the variety of cases provided a large data set, which favours the robustness and validity of the results [57].

### 2.1. Participants, Instruments and Data Collection

A total of 225 teachers, 84 men and 141 women, from 24 preschools and primary schools and 9 secondary schools in the north-west and central-west of Spain took part in this study. The age of the participants ranged from 26 to 64 years old. The average age of the teachers studied was 45.24 years, and the mode was 44 years. The teaching experience of the participants ranged from 1 to 39 years, with a mean of 19.2 years and a mode of 12 years. The length of time they had been working in their current school ranged from 1 month to 38 years. The average time working in the current school was 10.93 years, and the mode was 1 year. There were 66 teachers who held some kind of managerial or responsible position in their current school, while 159 did not.

Despite the fact that this is a qualitative research study, the levels that form the Primary and Secondary education stage in Spain have not been delimited. For this reason, a multiple case study was carried out with teachers who teach at any of the six levels of Primary Education (1st, 2nd, 3rd, 4th, 5th, and 6th levels) and the four levels of Secondary Education (1st, 2nd, 3rd, and 4th levels). For the selection of the teachers, all the schools in the Spanish autonomous communities of Galicia, Asturias, Castilla y León, and Extremadura were contacted. A meeting was held with the head teacher of the schools concerned to explain the research in detail. The head teacher informed the teachers about the research, and the teachers who wished to participate provided their e-mail address in order to receive the questionnaire. The choice of teachers for the study was therefore based on the availability of the school and teachers themselves. The delimitation of the participants for data collection was determined on the basis of the saturation of the results obtained and was closed when it was found that a larger number did not imply substantial variations.

The data collection instrument used was a structured non-standardised questionnaire developed ad hoc, as there was no other instrument on the market that was suitable for this research. In addition to profile data collection, the questionnaire consisted of 7 open questions and 23 items on a Likert-type scale (where 1—strongly disagree; 2—disagree; 3—neither agree nor disagree; 4—agree; and 5—strongly agree). These are psychometric instruments where the respondent must indicate their agreement or disagreement on a statement, or item, which is conducted through an ordered and unidimensional scale [58]. They are generally recognised as being among the most widely used instruments for measurement in the Social Sciences and Educational Sciences.

The questionnaire is structured in several blocks: the first one collects profile data. The second one aims to identify teachers’ perceptions of coexistence and bullying problems, while the last one focuses on questions about bullying prevention. The questionnaire was validated by 5 experts on the subject and on the type of research from 3 Spanish universities.

For its development, the researchers arranged an appointment with the head teacher of each school to present the research. Once the school had agreed to participate in the study and the anonymity of personal data was guaranteed, the participating teachers were informed about the research. Each of them was informed in advance of the voluntary nature of their participation in the research, as well as the purpose and use of the data collected, with these participants then giving their consent to participate.

Subsequently, in order to carry out the study, each of the schools provided e-mail addresses to which the questionnaire was then sent for dissemination among the teaching staff. The researchers were available to resolve any doubts or suggestions.

### 2.2. Data Analysis

In order to be able to examine the data collected through the questionnaires, it was necessary to organise all the information beforehand. The content analysis that was applied initially required an individualised treatment of the information obtained in each part of the questionnaire. On the one hand, the frequencies obtained in the different Likert-type scale items were counted, thus obtaining the absolute and relative frequencies. As for the open-ended questions, five experts (both in the subject matter under study and in content analysis) from four Spanish universities were consulted to define the main units of analysis (categories and subcategories). Content analysis was carried out using the Analysis of Qualitative Data (AQUAD) software version 7 (Tübingen, Baden-Wurtemberg, Germany). 

The choice of this software was based on its potential to interconnect the categories emerging from the teachers’ reflections with the conceptualisation and structuring process established by the researchers. Textual information from the questionnaires was coded using this programme. The AQUAD programme allows for a flexible and revisable process of continuous dialogue between the categories of analysis that emerge from the teachers’ own reflections and the effort of structuring and coherence. In this sense, a procedure was ensured that allowed the data to be coded with an appropriate interpretation. Content analysis was carried out by pairs of female researchers (with experience in this type of analysis) and in accordance with the experts’ indications.

The transfer of the results from AQUAD to the Excel software version 18 (Microsoft, Washington, Estados Unidos) was carried out in a systematised way. The strategies followed in the analysis have facilitated the presentation of the frequency count (absolute and relative) in the Section 3 Results. This contribution is interesting for being able to reflect the existence of some kind of predominance.

## 3. Results

This section presents the results of this study with some iconic and textual components. In order to optimise their organisation, they have been grouped into two sub-sections that answer the main objective and research questions.

### 3.1. Problems of School Coexistence: Bullying

The following sections show the results obtained in this study, focusing firstly on the teachers’ perceptions of the problem of school coexistence and then on bullying, identifying the traits of both the victim and the aggressor.

#### 3.1.1. Problems of School Coexistence

**Figure 1:**
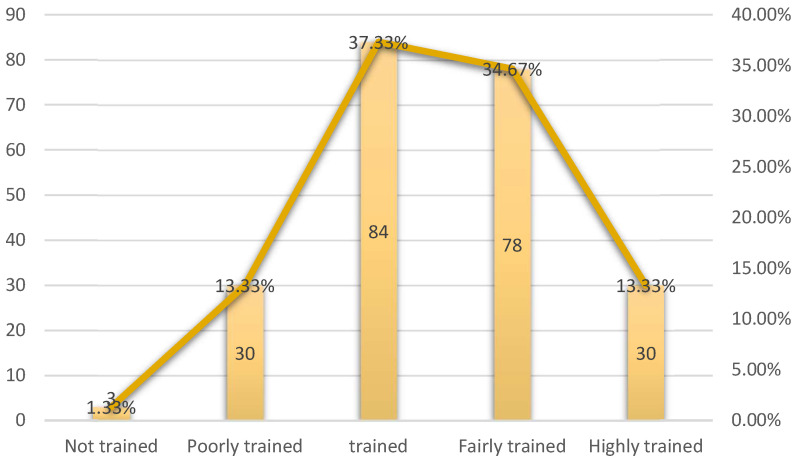
Teachers’ perceptions of their training on school coexistence problems.

This study shows that the teachers considered themselves to be sufficiently trained with regard to the problem of school coexistence (84/225; 37.33%). Some of the teachers involved in this study (78/225; 78%) said that they were fairly well trained. On the other hand, teachers who claimed to be poorly trained or highly trained were in the minority (30/225; 13.33%, respectively). Only 3 of the 225 teachers taking part in this study (1.33%) said that they were not trained at all (Figure 1).

More specifically, categorically speaking, it was found that teachers did not consider coexistence problems to have increased over the years, as only a few (72/225; 32%) strongly agreed with this. In fact, 63/225, or 28%, neither agreed nor disagreed (Table 1). However, more than half of the participants strongly agreed that coexistence problems had worsened with the use of ICT (117/225; 52%), and 51/225, or 22.67%, agreed. On the other hand, more than half of the teachers equated coexistence problems with those related to academic performance (123/225; 54.67%). However, 78/225, or 34.67%, considered most of the school’s coexistence problems to be of little relevance, indicating that there were no or few cases of bullying, compared to 66/225, or 29.33%, who disagreed. This shows the diversity of opinions among the teaching staff regarding the problems of school coexistence and, consequently, the lack of identification of these problems.

Of the 225 teachers, 57 (25.33%) completely agreed that the most problematic years in terms of coexistence were in the fifth or sixth grade of Primary School and third or fourth year of ESO. Fifty-four of the 225 teachers (24%) neither agreed nor disagreed. What the majority of teachers did agree on was that being a victim or perpetrator of bullying depended to a large extent on the family climate (64/225, or 28.44%, completely agreed, and another 64/225, or 28.44%, agreed).

Of the 225 teachers, 87 (38.67%) agreed that there were some cases of victims of coexistence problems that invited them to pay more attention. Also, teachers strongly disagreed that name-calling between students did not harm them (106/225; 47.11%). However, teachers neither agreed nor disagreed that a child who is bullied continuously is likely to commit suicide (87/225; 38.67%). This shows the lack of awareness of the consequences of a coexistence problem such as bullying and the importance of detecting it. It is also essential for children to have a good education of values. In this regard, 75 out of 225 teachers (33.33%) neither agreed nor disagreed that children, from primary school onwards, should be well trained in values.

On the other hand, teachers strongly disagreed that aggression and violent situations were a serious problem at the school (135/225; 60%) and that teachers themselves were attacked by students (141/225; 62.67%). Once again, the lack of awareness of problems of school coexistence and the inability to detect these situations at school is evident.

#### 3.1.2. Bullying: Victim and Bully Profile

**Figure 2:**
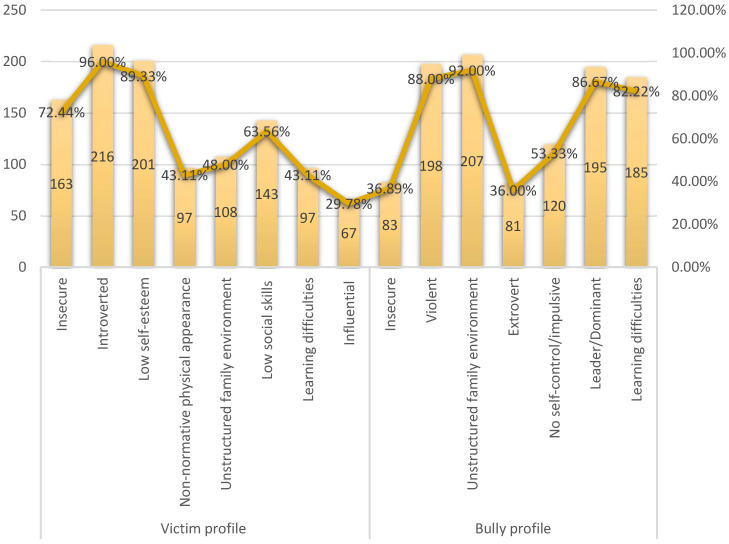
Profile of the bullying victim and bully.

Focusing on the coexistence problem of bullying, it is worth noting that the teachers identified the main profiles of both the victims of bullying and bullies (Figure 2).

Looking at the profile of the victim, almost all the teachers (216/225; 96%) claimed that they are introverted. A large percentage of teachers (201/225; 89.33%) indicated that a victim has low self-esteem. Similarly, 163 out of 225 teachers (72.44%) considered them to be insecure. To a lesser extent, they believed that they have low social skills (143/225; 63.56%). On the other hand, almost half of the teachers (108/225; 48%) were convinced that their family environment is unstructured. To a lesser extent, teachers highlighted other traits that a victim of bullying may have, such as a non-normative physical appearance and learning difficulties (97/225; 43.11% both). In addition, some teachers also considered them to have an impressionable personality (67/225; 29.78% both).

As an example, see the following extract:

*In general, and despite the fact that we can often be surprised by both victim and aggressor, and taking into account my experience, I would say that the victims of bullying are very introverted, insecure and have very low self-esteem. I think they sometimes have a non-normative physique, and maybe some learning difficulties as well. Unfortunately, sometimes they tend to have a dysfunctional family environment and they don’t have much family support (47-year-old teacher, state school, 6th grade Primary School. Lines of analysis 18–22).*

In relation to the profile of the bully, almost all the teachers (207/225; 92%) stated that they are pupils with a broken family environment. This trait is sometimes also present in the victims of bullying. A very high percentage of teachers (198/225; 88%) believed that they are violent people, while 195/225, or 86.67%, considered them to present leader/dominant traits. Also, a large proportion of teachers (185/225; 82.22%) said that aggressors suffer from learning difficulties, a trait again shared with victims. Slightly more than half of the teachers said that they are people without self-control and are impulsive (120/225; 53.33%). To a smaller extent, the other traits reported were insecurity (83/225; 36.89%) and extroversion (81/225; 36%).

An illustrative excerpt is presented below.

*I probably fall into some stereotype, but if I had to define a bullying aggressor, I would do it in the following way: he is a violent pupil, a bad student with learning difficulties and in most cases he brings problems from home, as his family is unstructured, with economic or social problems…He has a dominant personality, of a leader and he is often a leader. However, sometimes he is the most insecure boy in the whole group (57-year-old teacher, state school, 2nd grade Secondary School. Lines of analysis 34–39).*

### 3.2. Prevention of Bullying: Professionals, Programmes, and Preventive Measures

The results obtained in this study are shown below in terms of teachers’ perceptions of bullying prevention, as well as the professionals and programmes needed. The last section deals with the preventive actions carried out in schools.

#### 3.2.1. Prevention of Bullying

From this study, it appears that more than half of the teachers (131/225; 58.22%) strongly agreed that teachers should receive specific training to prevent bullying (Table 2). However, almost half of the participants (110/225; 48.89%) strongly agreed that teachers control their class and deal with bullying conflicts and aggressions not becoming a problem. This shows that, in spite of considering necessary training on bullying, they felt capable of solving the problems derived from bullying. Furthermore, they were aware that the teaching load prevents them from devoting more time to bullying problems (88/225, or 39.11%, agreed, and 84/225, or 37.33%, strongly agreed). In this sense, almost half of the teachers (103/225; 45.78%) strongly agreed that police action in schools was necessary to tackle bullying problems. However, 75/225, or 33.33%, of the teachers neither agreed nor disagreed that police talks in schools would be effective in terms of bullying prevention. This shows the importance they attach to intervention as opposed to prevention.

The participating teachers were not clear as to whether the work of surveillance during breaks would be effective in preventing bullying, with 54/225, or 24%, agreeing and 54/225, or 24%, disagreeing. The same was true for the idea of taking away mobile phones from pupils as an effective preventive measure against bullying, as 73/225, or 32.44%, agreed; 65/225, or 28.89%, strongly agreed; and 60/225, or 26.67%, strongly disagreed.

A part of the participants strongly agreed (73/225; 32.44%) that teachers’ actions in bullying cases are part of their educational tasks, whereas another part neither agreed nor disagreed (71/225; 31.56%). However, several teachers (51/225; 22.67%) completely disagreed with the statement that teachers were aware of the main successful bullying prevention programmes implemented in Spain. Along these lines, a large part of the sample (76/225; 33.78%) considered teachers, without the help of others, to not be prepared for problems related to bad relations and bullying.

#### 3.2.2. Professionals and Programmes for Bullying Prevention

This study shows that a large number of teachers (156/225; 69.33%) maintained that the Administration should allocate resources to make public job offers for professionals exclusively dedicated to coexistence problems such as bullying. On the other hand, 69/225, or 30.67%, did not consider it necessary.

**Figure 3:**
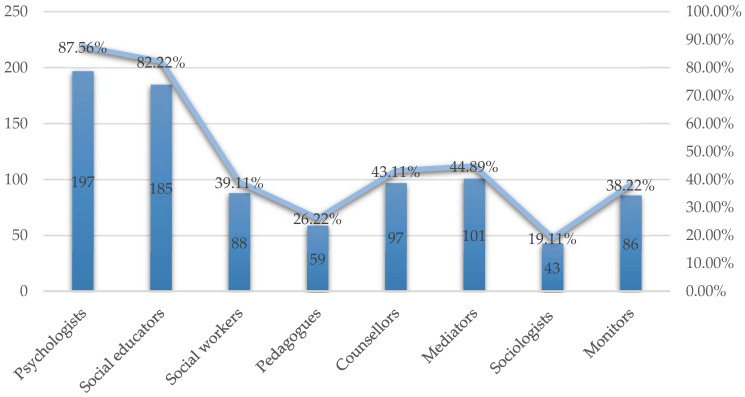
Professionals requested by teachers for the prevention of bullying.

The teachers who considered it necessary to implement the presence of professionals specialised in school problems such as bullying in schools mainly highlighted the figures of psychologists (197/225; 87.56%) and social educators (185/225; 82.2%) (Figure 3).

As an example, see the following extract:

*Of course, I believe that there should be professionals dedicated exclusively to student behaviour. The problems of school coexistence, especially bullying, are very serious problems. I think it would be very interesting to have a psychologist and one or more social educators in our school (53-year-old teacher, state school, 2nd grade Secondary School. Lines of analysis 43–48).*

**Figure 4:**
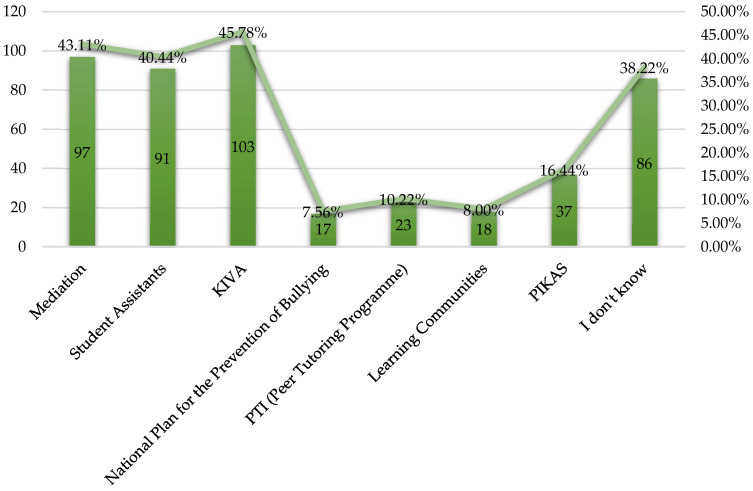
Preventive programmes for the prevention of bullying.

From this study, it appears that many of the teachers were not aware of many bullying prevention programmes. However, it is noteworthy that almost half of the teachers were aware of KIVA (103/225; 45.78%) (Figure 4). Several teachers also referred to the Mediation programme (97/225; 43.11%) and the Pupil Helper programme (91/225; 40.44%). Nevertheless, it is worth noting that a considerable number of teachers (86/225; 38.22%) stated that they were not aware of any bullying prevention programme.

The following illustrative extracts are presented:

*I do know some bullying prevention programmes, such as KIVA or Alumni Helper, but the truth is that we never put them into practice. (34-year-old teacher, state school, 1st grade Secondary School. Lines of analysis 41–43).*

*The truth is that I don’t know of any specific bullying prevention programme. We don’t have that here, we are a quiet school (61-year-old teacher, state school, 6th grade Primary School. Lines of analysis 52–55).*

#### 3.2.3. Preventive Measures Carried Out in Schools

**Figure 5:**
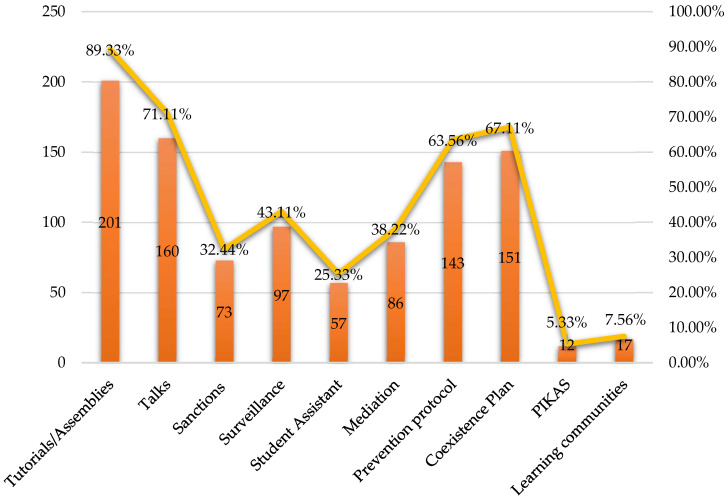
Preventive measures carried out in schools.

This study shows the preventive measures currently carried out by schools, as well as the preventive measures desired by the teachers themselves. In this sense, teachers indicated that they mainly carried out tutorials/assemblies (201/225; 89.33%) and talks (160/225; 71.11%) developed by teaching staff from the centre or from outside. To a lesser extent, they referred to what is stipulated in the Coexistence Plan (151/225; 67.11%) and the prevention protocol (143/225; 63.56%). Other measures included monitoring (97/225; 43.11%), mediation (86/225; 38.22%), sanctions (73/225; 32.44%), the Pupil Helper programme (57/225; 25.33%), learning communities (17/225; 7.56%), and the PIKAS programme (12/5.33%) (Figure 5).

These quotes are presented by way of example:

*In our school we deal with the issue of bullying mainly through tutorials and assemblies. Occasionally an external professional comes to give a talk to the whole centre or only to the last years of primary and ESO, it is very interesting (Teacher, 48 years old, State-subsidised school, 1st grade Secondary School. Lines of analysis 48–54).*

*We are working on learning communities with parent volunteers who improve coexistence, we have also applied for the board project on student helpers, we could do much more but there is a lack of hours to dedicate to this topic. (43-year-old teacher, state school, 2nd grade Secondary School. Lines of analysis 53–56).*

**Figure 6:**
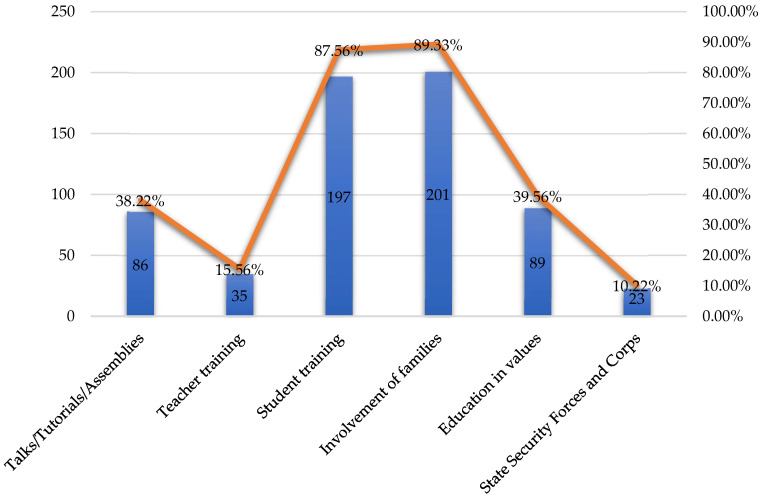
Preventive measures desired.

Having indicated the preventive actions carried out in their current schools, the teachers indicated their desired measures to prevent bullying. In this sense, teachers mainly believed it was necessary to involve families (201/225; 89.33%) and to train pupils (197/225; 87.56%) (Figure 6). To a lesser extent, they believed that a subject on education about values (89/225; 39.56%), talks/tutorials/assemblies (86/225; 38.22%), as well as teacher training (35/225; 15.56%) and the collaboration of the FCSE (State Security Forces and Corps) were of interest.

The following illustrative extracts are presented:

*Teacher training is essential: to know what the young people’s tools are and the strategies for action. I believe in training for all. I also find mediation programmes interesting, with training for students (51-year-old teacher, state-subsidised school, 3rd Secondary School. Lines of analysis 59–63).*

*I think that the main thing is the training of both pupils and teachers. The involvement of families is also necessary. We must continue with talks, tutorials…but education in values is necessary (Teacher, 39 years old, state-subsidised school, 5th Primary Education. Lines of analysis 62–65).*

## 4. Discussion

This section presents the discussion of the study. In order to optimise its organisation, it has been grouped into two sub-sections as well as results, answering the main objective and research questions.

### 4.1. Problems of School Coexistence: Bullying

According to the findings of this study, the teachers considered themselves to be sufficiently trained in the problems of coexistence at school. On the other hand, they did not perceive that coexistence problems had particularly increased over the years, but they did believe that ICT had had a negative influence. Previous studies point to cyberbullying as a new form of bullying supported by ICT [59]. In addition, teachers equated problems of coexistence with those related to academic performance. In this regard, previous research has shown that this form of aggression can have devastating consequences for both victims and perpetrators, affecting their mental health, academic performance, and social relationships [60,61].

The teachers downplayed the importance of coexistence problems at school, detecting few or no cases of bullying. They also did not believe that bullying could have dire consequences such as suicide. However, there is evidence establishing a relationship between exposure to bullying and the development of depression and suicide in children and adolescents [62,63].

From this study, it was clear that the teachers were certain of the importance of the family climate as well as of an education about values implemented in the early stages of education. In this sense, several works of research argue that the integration of diversity activities in schools reduces the incidence of bullying victimisation, and that improving the school climate is one of the most effective measures of reducing the rate of bullying [64,65,66].

This study found that the teachers identified the bullying victim as an introverted, insecure person, with low self-esteem, which coincides with studies such as that of [11,67]. The victim profile was established as sometimes lacking in social skills and coming from a dysfunctional family environment. They may have a non-normative physical appearance and learning difficulties [68]. In addition, some teachers also considered them to have an impressionable personality.

In relation to the profile of the bully, the teachers maintained that they are pupils with a broken family environment. They are violent, impulsive, lacking in self-control, and have leader/dominant traits. Previous studies show that the aggressor insults, criticises, and ridicules one or more classmates through inappropriate comments and insinuations, using a superior or threatening tone [69,70]. The teachers also reported that bullies suffer from learning problems. According to previous findings [71], bullies tend to have poor academic performance scores, a result that is also confirmed by this current study.

### 4.2. Prevention of Bullying: Professionals, Programmes and Preventive Measures

In this study, it was discovered that teachers found the idea of receiving specific training to prevent bullying interesting. However, they believed that they were capable of controlling their class and tackling conflicts arising from bullying, considering their actions as part of their educational tasks. In this sense, insufficient bullying prevention mechanisms and a lack of awareness of educators about protection from student bullying are crucial factors contributing to the occurrence of bullying [72,73].

They stated that they could not devote time to bullying problems because of their teaching load. In fact, they considered police action necessary to tackle bullying problems—not so much their talks in schools. They were not clear about the necessary preventive measures and did not have much knowledge of the main successful bullying preventive programmes applied in the Spanish context. Some research [74,75] highlights the need for teachers to receive psychoeducational intervention training in order to teach their students problem-solving skills, which can redirect potentially negative or passive behaviours towards positive problem-solving and leadership skills.

In fact, without the help of others, he/she would not see him/herself ready to solve bullying problems. In this sense, it is important to underline the important role of governments and public authorities in preventing a problem that harms children in schools on a daily basis [76,77,78]. Along these lines, many teachers argued that the Administration should allocate resources to create public job offers for professionals that are exclusively dedicated to coexistence problems such as bullying, highlighting the experience of psychologists and social educators.

This study showed that many of the teachers were not aware of many bullying prevention programmes, with some highlighting the KIVA programme. Along these lines, in the study by [79], they analysed more than 1200 schools implementing the KIVA programme for 6 years, finding that the success rate of the intervention was higher in primary school grades than in secondary school. In some cases, they knew of no programme at all. In reality, the preventive measures carried out are mainly focused on tutorials/assemblies and talks. Sometimes they refer to the Plan de Convivencia, the prevention protocol, or a programme such as *Alumno Ayudante* or PIKAS. It should be noted that comprehensive studies [21,80] have demonstrated the effectiveness of these bullying prevention programmes in reducing both bullying and victimisation by approximately 20%.

The findings of this research indicated that the teachers believed that family involvement and student education were necessary as preventive measures. Along these lines, school-based anti-bullying programmes that include a parent component result in small but significant reductions in bullying [81,82]. The teachers also advocated a subject on values education, talks/tutorials/assemblies, teacher training, and collaboration with law enforcement agencies. Some studies have shown that the inclusion of moral education in the curriculum, together with the implementation of cultural activities, proved to be an effective measure to prevent bullying [65]. Other studies show that schools that implemented bullying prevention and control measures showed a marked decrease in bullying incidents compared to those that did not [83,84,85]. These findings suggest that bullying prevention and control measures could effectively reduce bullying incidents among students.

## 5. Conclusions

The teachers considered themselves to be well trained with regard to the problems of school coexistence. However, the findings of this study indicate that they are not fully aware of the consequences of these problems. The teachers themselves downplayed the importance of these problems and were not able to identify them at school. However, they were able to identify the profile of both the victims of bullying and the aggressors.

As far as bullying is concerned, this study shows that although the teachers considered training on the subject to be necessary, they believed they were capable of solving the problems arising from it. They also showed a preference for intervention rather than prevention, which makes it difficult to eradicate bullying. Moreover, they showed little knowledge of preventive programmes and measures. They were able to identify social educators and psychologists as the appropriate professionals to exclusively deal with coexistence problems such as bullying. The prevention programmes they were most familiar with were KIVA and Mediation. As preventive actions, they carried out tutorials/assemblies and talks. The preventive measures that they desired should mainly focus on the involvement of families and the training of pupils.

This research underlines the need for awareness raising and the training of teachers, students, and families on bullying issues. These findings enhance our understanding of the important role of the educational community in school issues, especially bullying, and reinforce the need to work on prevention and intervention through preventive programmes and actions.

## 6. Limitations and Future Directions

This work has several limitations that future researchers should address. Firstly, despite being a mixed study, it is heavily dominated by the qualitative part. Also, all data came from the participants’ questionnaires. On the other hand, the information was collected at the time of the study, which makes it difficult to know whether there is a problem of school coexistence after the study. Despite these limitations, this research supports the enrichment of in-service teacher studies and provides a basis for more rigorous research designs in the future.

In general, there are likely to be several possible directions for future researchers. Firstly, future researchers could consider purely quantitative research methods. Also, other instruments could be used for data collection. In addition, researchers may employ longitudinal examinations, studying school coexistence problems in schools over a long period of time. Other stakeholders, such as families and pupils, and also those from other countries, could be involved in research. Different types of schools—public, charter, and private—could be differentiated, and a comparative analysis could be made.

## Figures and Tables

**Table 1:** Teachers’ perceptions of the problems of school coexistence.

	Teachers (n = 225)									
	fi	n_i_	fi	n_i_	fi	n_i_	fi	n_i_	fi	n_i_
Items	1		2		3		4		5	
Problems of coexistence have increased over the years.	18	8.00%	21	9.33%	63	28.00%	51	22.67%	72	32.00%
Problems of coexistence have worsened with the use of ICT.	6	2.67%	15	6.67%	36	16.00%	51	22.67%	117	52.00%
Problems of coexistence are as important as those related to academic performance.	6	2.67%	21	9.33%	33	14.67%	42	18.67%	123	54.67%
Is there a case of a victim of coexistence problems that calls for more attention?	15	6.67%	27	12.00%	45	20.00%	87	38.67%	51	22.67%
A child who suffers continuous bullying is likely to commit suicide.	12	5.33%	45	20.00%	87	38.67%	51	22.67%	30	13.33%
Children, from primary school onwards, are well trained in values.	21	9.33%	54	24.00%	75	33.33%	60	26.67%	15	6.67%
Name-calling among students does not hurt them.	106	47.11%	49	21.78%	43	19.11%	18	8.00%	9	4.00%
Assaults and violent situations are a serious problem in the centre.	135	60.00%	63	28.00%	27	12.00%	0	0.00%	0	0.00%
Teachers themselves come under attack from pupils.	141	62.67%	54	24.00%	18	8.00%	9	4.00%	3	1.33%
Most of the school’s coexistence problems are of little relevance; there are no or few cases of bullying.	21	9.33%	66	29.33%	36	16.00%	24	10.67%	78	34.67%
The most problematic grades in terms of coexistence are the fifth or sixth year of Primary School and third or fourth year of ESO.	27	12.00%	51	22.67%	54	24.00%	36	16.00%	57	25.33%
Being a victim or perpetrator of bullying depends to a large extent on the family climate.	24	10.67%	27	12.00%	46	20.44%	64	28.44%	64	28.44%

Note: fi = absolute frequency; n_i_ = relative frequency. 1: Strongly disagree; 2: Disagree; 3: Neither agree nor disagree; 4: Agree; 5: Strongly agree.

**Table 2:** Teachers’ perceptions of bullying prevention.

	Teachers (n = 225)									
	fi	n_i_	fi	n_i_	fi	n_i_	fi	n_i_	fi	n_i_
Items	1		2		3		4		5	
Teachers should receive specific training to prevent bullying.	0	0.00%	6	2.67%	18	8.00%	70	31.11%	131	58.22%
Teachers control their class and deal with bullying conflicts and aggressions not becoming a problem.	0	0.00%	6	2.67%	21	9.33%	88	39.11%	110	48.89%
Teachers, without the help of others, are not prepared for problems of bad relations and bullying.	15	6.67%	33	14.67%	54	24.00%	76	33.78%	47	20.89%
Police talks in schools are effective in terms of bullying prevention.	54	24.00%	42	18.67%	75	33.33%	42	18.67%	12	5.33%
The teaching load prevents teachers from devoting more time to bullying problems.	3	1.33%	15	6.67%	35	15.56%	88	39.11%	84	37.33%
Teachers are aware of the main successful bullying prevention programmes implemented in Spain.	51	22.67%	48	21.33%	48	21.33%	48	21.33%	30	13.33%
Monitoring during breaks is effective in preventing bullying.	45	20.00%	54	24.00%	45	20.00%	54	24.00%	27	12.00%
Confiscating mobile phones from pupils is an effective preventive measure against bullying	60	26.67%	12	5.33%	15	6.67%	73	32.44%	65	28.89%
Police action at school is necessary to tackle bullying problems.	18	8.00%	15	6.67%	49	21.78%	40	17.78%	103	45.78%
Teachers’ actions in cases of bullying are part of their educational tasks.	18	8.00%	12	5.33%	71	31.56%	51	22.67%	73	32.44%

Note: fi = absolute frequency; n_i_ = relative frequency. 1: Strongly disagree; 2: Disagree; 3: Neither agree nor disagree; 4: Agree; 5: Strongly agree.

## Data Availability

Restrictions apply to the datasets: the data are part of an ongoing study and due to technical limitations.
